# Population Control of Upconversion Energy Transfer for Stimulation Emission Depletion Nanoscopy

**DOI:** 10.1002/advs.202205990

**Published:** 2023-04-23

**Authors:** Yongtao Liu, Shihui Wen, Fan Wang, Chao Zuo, Chaohao Chen, Jiajia Zhou, Dayong Jin

**Affiliations:** ^1^ Smart Computational Imaging Laboratory (SCILab) School of Electronic and Optical Engineering Nanjing University of Science and Technology Nanjing Jiangsu Province 210094 P. R. China; ^2^ Institute for Biomedical Materials and Devices (IBMD) Faculty of Science University of Technology Sydney Sydney NSW 2007 Australia; ^3^ School of Physics Beihang University Beijing 102206 P. R. China; ^4^ School of Electrical and Data Engineering Faculty of Engineering and Information Technology University of Technology Sydney Sydney NSW 2007; ^5^ UTS‐SUStech Joint Research Centre for Biomedical Materials & Devices Southern University of Science and Technology Shenzhen Guangdong 518055 P. R. China

**Keywords:** nonlinear optics imaging, stimulated depletion emission microscopy (STED), super‐resolution imaging, upconversion nanoparticles

## Abstract

Upconverting stimulated emission depletion microscopy (U‐STED) is emerging as an effective approach for super‐resolution imaging due to its significantly low depletion power and its ability to surpass the limitations of the square‐root law and achieve higher resolution. Though the compelling performance, a trade‐off between the spatial resolution and imaging quality in U‐STED has been recognized in restricting the usability due to the low excitation power drove high depletion efficiency. Moreover, it is a burden to search for the right power relying on trial and error as the underpinning mechanism is unknown. Here, a method is proposed that can easily predict the ideal excitation power for high depletion efficiency with the assistance of the non‐saturate excitation based on the dynamic cross‐relaxation (CR) energy transfer of upconversion nanoparticles. This allows the authors to employ the rate equation model to simulate the populations of each relevant energy state of lanthanides and predict the ideal excitation power for high depletion efficiency. The authors demonstrate that the resolution of STED with the assistance of nonsaturated confocal super‐resolution results can easily achieve the highest resolution of sub‐40 nm, 1/24^th^ of the excitation wavelengths. The finding on the CR effect provides opportunities for population control in realizing low‐power high‐resolution nanoscopy.

## Graphical abstract

This study presents a novel method for predicting the ideal excitation power in upconverting STED microscopy to achieve high depletion efficiency and resolution. By using the dynamic cross‐relaxation energy‐transfer modeling, the researchers can simulate each relevant energy state of lanthanides and predict the ideal excitation power. Results demonstrate 33‐nm resolution, offering new opportunities for low‐power high‐resolution nanoscopy.

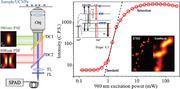

## 1 Introduction

Stimulated emission depletion (STED)^[^ ^1^ ^]^ microscopy is a fast super‐resolution imaging technique without the requirement of postreconstruction.^[^ ^2^, ^3^ ^]^ STED employs a doughnut‐shaped beam to quench fluorophores from the peripheral area of the emission spot through stimulated emissions. A gaussian‐shaped beam overlaps with the doughnut beam to excite the nonquenched fluorophores inside the center of the doughnut. Its image resolution is linear to the radius of the nonquenched area, in which a smaller radius provides a better resolution. High power of depletion beam (e.g., 100 mW of 775 nm laser,^[^ ^4^ ^]^ or 90 mW of 620 nm laser^[^ ^5^ ^]^) has been required to reduce the size of the nonquenched area. However, high depletion power causes photobleaching and phototoxicity, which decreases the biocompatibility of the technology.^[^ ^6^, ^7^ ^]^

Upconverting stimulated emission depletion (U‐STED) microscopy uses a library of upconversion nanoparticles (UCNPs) to reduce the requirement in depletion power for high‐resolution imaging.^[^ ^8^, ^9^, ^10^ ^]^ With the dopants of lanthanide sensitizers and emitters, the upconversion nanoparticles can absorb near‐infrared photons, transfer the energy to high excited states, and produce short‐wavelength emissions.^[^ ^11^, ^12^, ^13^ ^]^ The multiple real intermediate states of the lanthanides enable the low power requirement for a stimulated depletion process. For example, in a high concentration Tm^3+^‐doping system, the interionic cross‐talk induces a photon avalanche (PA)‐like effect, in which a population inversion happens between the intermediate state and the ground state within a single nanoparticle.^[^ ^8^, ^14^ ^]^ By a trigger of 808 nm doughnut laser, the PA‐like effect at the intermediate state can partially interrupt the upward upconversion pathways and facilitate depletion of the high excited states. By exploring this mechanism, in 2017, our group achieved a sub‐30 nm resolution image with a STED beam density of two orders of magnitude lower than that used on fluorescent dyes.^[^ ^8^ ^]^ Zhan's group further demonstrated the successful resolving of microtubules with an 80‐nm resolution using U‐STED.^[^ ^9^ ^]^

In the U‐STED operation, a key condition is to find out the appropriate excitation power, which makes a balance between the depletion efficiency and imaging quality, i.e., signal‐to‐noise ratio. Till now, finding the appropriate excitation power has been an empirical and laborious process, relying on trial and error due to the unknown underlying mechanism. Despite the obvious general understanding, i.e., the excitation power‐dependent nonlinear responses for electron‐generation rates and populations at each energy level in an upconversion system play an important role in determining the efficiency of depletion,^[^ ^15^, ^16^, ^17^ ^]^ a clear clue for a precise experimental operation is absent. This is because of the limited understanding regarding the intricate energy‐transfer process in a nanoparticle, which has thousands of lanthanides doped in the lattice and the variable doping concentration further complicate the energy‐transfer network.

In this work, we revisit the classical U‐STED system with the Yb^3+^ and Tm^3+^ dopants in the *β*‐NaYF_4_ nanoparticles. We explore that a ground‐state‐involved cross‐relaxation among the Tm^3+^ emitters shows a dynamic response to the excitation power and therefore quantitatively determine the depletion efficiency. This relationship is predictable by theoretical model analysis using steady‐state rate equations. By measuring the power‐dependent intensity curve for blue emission centered around a wavelength of ≈455 nm, we observe a threshold of a slope jump, which serves as the point for the ideal power value used in the U‐STED experiment. By fixing the excitation power, we investigate the evolution of the depletion power‐efficiency relationship for the intermediate energy state of Tm^3+^. We find that the low excitation power results in a sweet spot where the depletion rate surpasses the pumping rate while achieving the population inversion. By controlling the doping concentration, we study the effect of the upconversion rate on depletion efficiency, indicating an optimized doping concentration for U‐STED. We, therefore, demonstrate that a moderate condition of 1 mW excitation power and 27 mW of depletion power is sufficient to achieve 33 nm resolution. Our study suggests a scope to reduce the power in high‐resolution imaging.

## 2 Experiment and Results

### 2.1 Proposed Mechanism and Numerical Simulation of U‐STED

**Figure 1:**
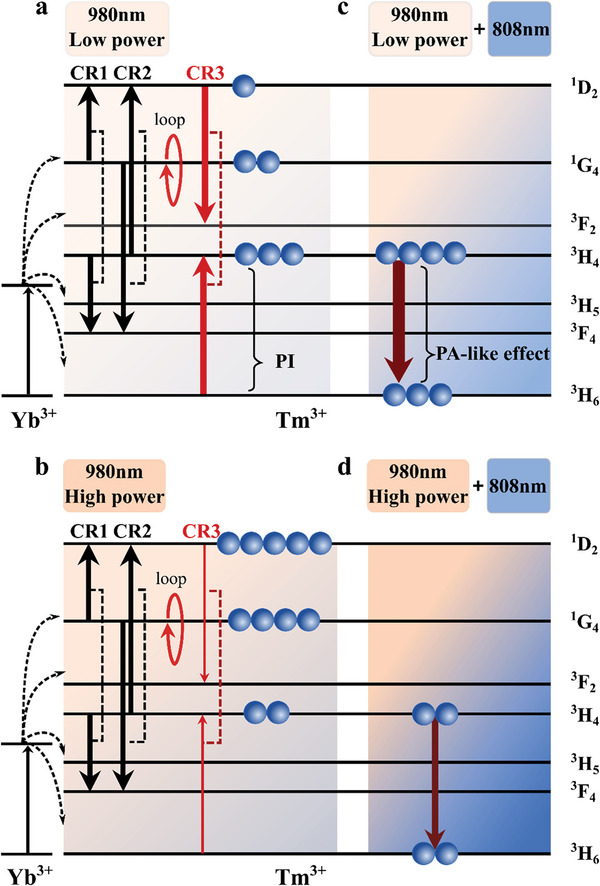
The schematic of dynamic cross‐relaxation rate depletion mechanism in the photon avalanche‐like process under low and high excitation powers. a,b) The energy level diagram of Tm^3+^ in *β*‐NaYF_4_ shows the main cross‐relaxation under low (a) and high (b) excitation power of 980 nm. c,d) With a fixed power of depletion laser beam of 808 nm, the electron distributions of Tm^3+^ under the low (c) and high (d) excitation power of 980 nm. CR1, CR2, and CR3 refer to cross‐relaxation processes that occur between (^1^G_4_, ^3^H_4_,^1^G_4_, ^3^H_4_) and (^1^D_2_, ^3^H_6_) states at both the ground and upper excited levels, leading to population distribution of (^1^D_2_,^3^F_4_,^3^F_4_,^1^D_2_) and (^3^F_2_,^3^H_4_), respectively.

**Figure 2:**
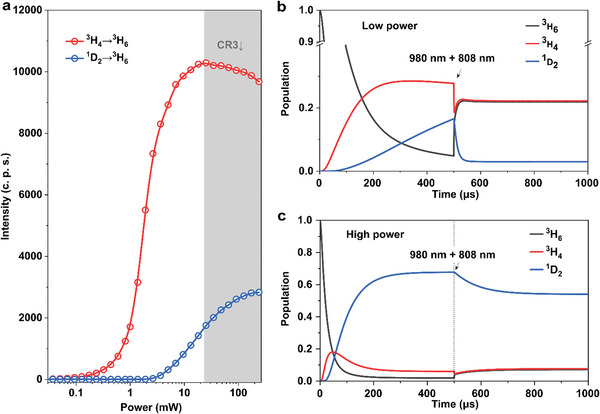
Numerical simulation for the 8% doped UCNPs under dual‐laser excitation with a dynamic CR3. a) The power‐dependent transition response of the upconverted emission was measured at 455 and 800 nm. b,c) By applying a fixed power of depletion laser beam of 808 nm after 500 µs, the numerical simulation results of the emitter (Yb^3+^–Tm^3+^) population distribution under low (b) and high (c) excitation power of 980 nm.

**Figure 3:**
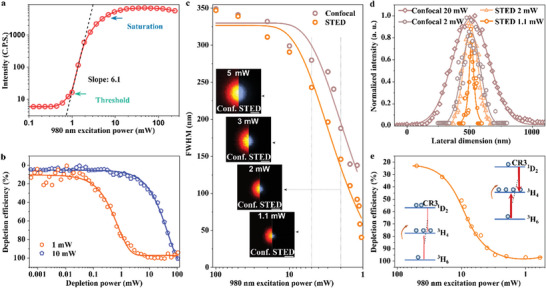
The PSFs of confocal and U‐STED under different excitation power. a) The emission from the UCNPs at *λ*  =  455 nm exhibits a strong super‐linear dependence on the excitation power. b) Depletion efficiency of the upconversion emission at 455 nm, which is measured by a fixed excitation power of 980 nm laser overlap with a continuously tunable depletion laser of 808 nm. The power density for 1 mW excitation laser is 0.27 MW cm^−2^. c) The full width at half maximum of confocal with U‐STED enhancement obtained with decrease excitation power. The depletion power is fixed at 27 mW (3.4 MW cm^−2^) for all the results. Pixel dwells time, 3 ms; d) The cross‐section profiles of the selected points of single particles. e) Depletion efficiency of UCNPs as a function of the excitation power. Most of the spectroscopic studies on the excitation and depletion processes can be referenced in our previous works.^[^ ^8^ ^]^

As illustrated in **Figure** **1**, the simplified energy level diagram shows the energy‐transfer processes between the sensitizer Yb^3+^ ions and the emitter Tm^3+^ ions with high concentration (i.e., >2 mol%). We propose the dominant dynamics among the Tm^3+^ energy levels under 980 nm laser excitation as follow: (1) a power‐dependent cross‐relaxation (CR3:^1^D_2_,^3^F_2_‐^3^H_6_,^3^H_4_) happens between the ground state and the upper‐excited levels followed by CR1 (^1^G_4_,^1^D_2_‐^3^H_4_,^3^F_4_) and CR2 (^3^H_4_,^1^D_2_‐^1^G_4_,^3^F_4_) to populate the ^1^D_2_ level (Figure 1a); (2) the higher excitation power results in the lower probability of CR3 (Figure 1b); (3) with the applying of a depletion beam at 808 nm (Figure 1c), the photo avalanche‐like depopulation happens at ^3^H_4_ level as suggested in our previous work,^[^ ^8^ ^]^ hindering the population for upward higher energy levels; (4) while raising the 980 nm power at the two beam condition (Figure 1d), the photo avalanche cannot happen effectively and all the upward upconversion transitions are still obvious. Therefore, it is challenging to achieve high‐efficiency STED imaging by collecting the emission from ^1^D_2_ level.

In our proposed mechanism, a critical point is the power‐dependent variable transition rates of the ground‐state‐involved CR3, which determines the population inversion of ^3^H_4_ versus the ground state. To verify the variable CR3 process, we measured the power‐dependent emission at 455 nm from ^1^D_2_ level and 800 nm emission from ^3^H_4_ level (**Figure** **2**a). The downward trend of 800 nm emission intensity at the high excitation power indicates the declined CR3 while the 455 nm emission keeps increasing.

By neglecting minor cross‐relaxations and nonradiative relaxation pathways, we embed the feature of dynamic CR3 rates into a numerical equation model to simulate the transient response for the 8 mol% Tm^3+^‐doped UCNPs under dual‐laser excitation (during the first 500 µs, 980 nm laser used as single excitation beam and then coupling 808 nm laser as the depletion beam). The simulation details are shown in Note S1 (Supporting Information), with Table S1 (Supporting Information) summarizing the yielded rate parameters. The simulation results plotted in Figure 2b,c help to visualize the electron population evolutions of the key energy levels.

Low excitation power of 980 nm laser with the 808 nm depletion beam at a fixed power results in a high depletion efficiency (Figure 2b). In contrast, the high excitation power shows a relatively low depletion efficiency (Figure 2c). The differences shown above between the two excitation power conditions indicate that the critical population distribution in ^3^H_4_ and ^1^D_2_ is driven by a key mechanism related to the cross‐relaxation rate (CR3). The large CR3 formed a positive feedback process to accelerate the populations in the intermediate excited state of ^3^H_4_, which is the precondition for the photon avalanche.^[^ ^8^, ^18^ ^]^ And the cross‐relaxation coefficient surpasses the intrinsic decay rate from the ^1^D_2_‐excited level induce a high depletion efficiency.

### 2.2 Single and Dual‐Laser Confocal Microscopic Study

To validate our hypothesis and simulation, a home‐made dual‐laser super‐resolution system with confocal imaging function is established to experiment. Figure S2 (Supporting Information) provides a schematic illustration of an UCNPs STED nanoscopy system. In the U‐STED nanoscopy, a 980 nm laser is used to excite the UCNPs and produce an ordinary diffraction‐limited focus. The excitation laser is followed by an donut‐808 nm laser that is used to deplete the upconverion emission (455 nm) at the periphery of the excitation focus by a photon avalanche‐like process.^[^ ^8^, ^19^, ^20^ ^]^ The intense cross‐relaxation between the excited energy and lower energy level accelerates the accumulation of emitters in the intermediate excited state. Hence, the depletion efficiency is sensitive to the electron distribution in the intermediate excited state. To investigate the electron distribution in highly doped upconversion nanoparticles (NaYF_4_: 20% Yb^3+^, 8% Tm^3+^) and its effects, the power‐dependence curve of 455 nm (four‐photon excitation process) emission was measured, as shown in **Figure** **3**a. The threshold in the power‐dependence curve is the trigger point for the population process in the energy state of 455 nm excited state (^1^D_2_). When the populations at this state obtained a dynamic equilibrium between intermediate state and excited state, the saturation point will appear. The cross‐relaxation effect in high‐doping particles changes the balance between the different energy level redistribution processes, which results in a steeper excitation‐emission curve, with a maximum slope of 6.1, as shown in Figure 3a. The evolution indicates that the electron distribution at each energy level could be controlled by the excitation power. And the depletion efficiency will be substantially affected by the population distribution.

To investigate the electron population‐dependent depletion efficiency, we added the depletion beam of 808 nm to the system. By using the different excitation power of 980 nm laser with fixed depletion power (27 mW) of 808 nm laser, a depletion efficiency curve was obtained as shown in Figure 3b, which was measured by two overlapped Gaussian beams. The threshold power of 1 mW and saturation power of 10 mW result in a significantly different population in the intermediate state. The higher depletion efficiency of 1 mW excitation power is benefiting from the high CR3, in which an easy depletion electron configuration is formed. Inversely, the excitation power around the saturation point applies a low CR3 and induces a high pumping population rate in the upper‐excited state, so a higher depletion power was needed to achieve the equivalent effect.

### 2.3 Depletion Process in U‐STED

To further investigate the depletion process in U‐STED, we inspect the point spread functions (PSF) and depletion efficiency. Figure 3c shows the comparison of PSFs by confocal scanning and U‐STED. According to the power‐dependence curve (Figure 3a), smaller excitation power brings the photoresponse of UCNP close to the threshold point or the region with the highest nonlinearly, leading to a reduced FWHM in PSF for confocal scanning^[^ ^21^, ^22^, ^23^, ^24^ ^]^ (see Figure S3, Supporting Information, for full PSFs under different excitation power). Notably, the confocal scanning can directly provide super‐resolution, with 126.4 nm resolution (exceeds the diffraction limit by a factor of two) for 1.1 mW of excitation power. Strikingly, the FWHM for U‐STED decreases with excitation as shown at their images (inset, Figure 3c) and cross‐section profiles (Figure 3d). This result indicates both the pumping rate and the depletion rate at the intermediate state nonlinearly respond with the changing of excitation power. The nonlinearity comes from the fact that the electron‐generation rate in the upconversion system depends on the electron population. The depletion efficiency for different excitation power (Figure 3e) examines the depleted population with a response to the total population at the intermediate state. With high excitation power, the small CR3 induces a lower electron‐generation rate in the intermediate state than the upper‐excited state, which leads to a lower depletion efficiency than that for the low excitation power condition. As the decreasing of the excitation power, both the cross‐relaxation rate and the relative electron population in the intermediate decrease, which leads to striking enhancement for the depletion efficiency. When the excitation power decreases to a sweet spot where the electron depletion rate is close to the generation rate and the rate of CR3 tends to be a stable value, the depletion efficiency becomes “saturated” with a maximum of 97%. Note here we fixed the depletion power as 27 mW for a fixed depletion rate. This power is the minimum to achieve the optimized depletion efficiency (see Figure 3b).

### 2.4 Emitter‐Doping Modulation at the Depletion Process

**Figure 4:**
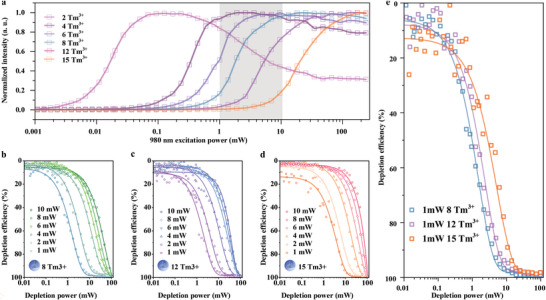
The depletion efficiency with respect to Tm‐doping concentration. a) The power‐dependence curve of 800 nm emission from the UCNPs with different doping concentrations. b–d) The depletion efficiency of the UCNPs with 8% Tm^3+^ (b) 12% Tm^3+^ (c), and 15% Tm^3+^ (d) at different excitation power. e) Comparison of the depletion efficiencies with different doping concentrations at the same excitation and depletion power.

Apart from the excitation power, the electron distribution is also related to the doping concentration of Tm^3+^. From our previous work,^[^ ^8^ ^]^ higher doping concentration will reduce the average distance of emitters, leading to intense cross‐relaxation and population inversion at the intermediate state, resulting in higher depletion efficiency. **Figure** **4**a shows the power‐dependence curve of 800 nm emission from the UCNPs with different doping concentrations. The decreasing tendency at higher excitation power further validates our proposed mechanism of the dynamic cross‐relaxation rate.Figure 4b–d shows the depletion efficiencies for UCNPs with Tm^3+^‐doping concentrations of 8%, 12%, and 15%, respectively. As decreasing the excitation power from 10 to 1 mW, the depletion efficiency is enhanced, which confirms the depleting processes are similar for all the UCNPs with high Tm^3+^ concentrations. While the 15% doping introduces the most obvious difference in depletion efficiency between 1 and 10 mW excitations. This is because the high doping concentration nonlinearly enhances the photon conversion and emission process at higher excitation power, and the resultant high electron‐generation rate will reduce the depletion efficiency. We further compared the depletion efficiencies for 8%, 12%, and 15% under 1 mW excitation power in Figure 4e, where 8% performs the best when the depletion power is higher than 1 mW. This result suggests that once the doping concentration is enough to generate population inversion, a larger doping concentration does not help the depletion. Because extreme high doping increases the photon conversion efficiency, which increases the electron‐generation rate at a higher state.^[^ ^19^, ^20^ ^]^ These electrons at higher states cannot be directly depleted, which causes an inferior depletion efficiency.

### 2.5 Imaging of UCNPs with the Optimized Condition

**Figure 5:**
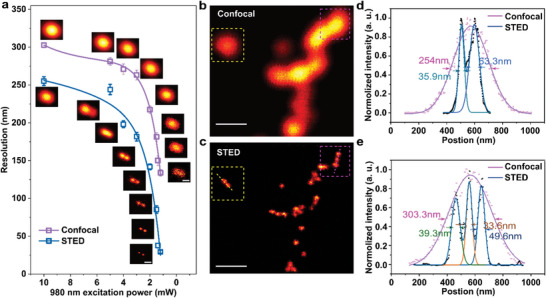
Super‐resolution image of the upconversion nanoparticles. a) The resolution of confocal and STED enhancement obtained with a decrease of excitation power. The depletion power we keep at 3.4MW cm^−2^. Pixel dwell time is 3 ms. Insets, confocal, and STED image at corresponding power points. b,c) Confocal and super‐resolution images of the 39 nm 8% Tm^3+^‐doped UCNPs. Pixel dwells time, 2 ms; scale bar 500 nm. Dashed boxes mark an area containing closely spaced UCNPs that can be resolved in upconversion‐STED but not in confocal imaging. d,e) Intensity profiles between the arrows across the UCNPs in confocal (b) and STED (c). The best FWHM is 33.6 nm after Gaussian fitting. In this experiment, 8% Tm^3+^‐doped UCNPs with the diameters of 39 nm is selected as the imaging particles (see in Supporting Information Note S5). The excitation power of confocal is 10 mW. The results in c, d, and e are after deconvolution.

We demonstrate the super‐resolution imaging of UCNPs by both confocal microscopy and U‐STED. **Figure** **5**a shows the images of a pair of UCNPs with spacing about 118 nm. As decreasing of the excitation power, both confocal microscopy and U‐STED enhances their resolution, achieving FWHM of 126.4 and 40.6 nm at 1 mW. Figure 5b,c shows the confocal image and the optimized U‐STED of free dispersed UCNPs, respectively. With the optimized condition, the U‐STED clearly shows us the particles distribution with the distance under diffractive limit. The cross‐line profiles of the selected areas (marked by the yellow and red box) are shown in Figure 5d,e, respectively. The best achievable FWHM is around 33 nm, 1/30^th^ of the excitation wavelength.

## 3 Conclusion

In conclusion, we investigated the roles of electron population generation and distribution in the depletion process for U‐STED. We found that the critical population in the intermediate state is driven by a key dynamic cross‐relaxation rate (CR3), which is related to the excitation power. A lower excitation power leads to a higher cross‐relaxation between the ground state and the upper‐excited levels, which effectively enhances the depletion efficiency. By embedding the dynamic CR3 into a numerical rate equation model, we simulated that the depletion efficiency is sensitive to the electron distribution in the intermediate state that is driven by the dynamic CR3. We further verify this dynamic response of cross‐relaxation by the decreasing tendency of a series of power‐dependence curves at high excitation power. A trigger point is implemented as a threshold at the power‐dependence curve to achieve the highest depletion efficiency. Benefitting from this sweet spot of the excitation power, the confocal image can directly provide super‐resolution results under a steeper excitation‐emission curve with a slope of 6.1. We further demonstrate the depletion efficiency affected by the doping concentration. The doping concentration of 8% Tm^3+^ is the optimized concentration for U‐STED. Finally, we demonstrate the super‐resolution imaging of UCNPs by both confocal microscopy and U‐STED under the excitation threshold point. The resolution of U‐STED and confocal can achieve an extremely high resolution around 33 and 126.4 nm at 1 mW, respectively. Hence, this work provides a way to increase the resolution and signal‐to‐noise ratio for U‐STED. In addition, the understanding of the energy‐transfer competition in UCNPs from this work will benefit a series of upconverting super‐resolution imaging technologies including Upconversion Nonlinear Structured Illumination Microscopy (U‐NSIM),^[^ ^25^ ^]^ multiphoton upconversion time‐encoded structured illumination microscopy (MUTE‐SIM)^[^ ^26^ ^]^ and near‐infrared emission saturation (NIRES) nanoscopy.^[^ ^27^, ^28^ ^]^

## Conflict of Interest

The authors declare no conflict of interest.

## Supporting information

Supporting Information

## Data Availability

The data that support the findings of this study are available from the corresponding author upon reasonable request.
