# Supplementary material for: Population Control of Upconversion Energy Transfer for Stimulation Emission Depletion Nanoscopy
Source: Adv Sci (Weinh). 2023 Apr 23;10(20):2205990. doi: 10.1002/advs.202205990 (PMC10369235; doi:10.1002/advs.202205990)
Supplement: Supplementary file 1 — Supporting Information [file ADVS-10-2205990-s001.pdf]

ADVANCED SCIENCE
Open Access

Supporting Information

for *Adv. Sci.*, DOI 10.1002/advs.202205990

Population Control of Upconversion Energy Transfer for Stimulation Emission Depletion Nanoscopy

Yongtao Liu, Shihui Wen, Fan Wang, Chao Zuo, Chaohao Chen, Jiajia Zhou* and Dayong Jin**

Population control of upconversion energy transfer for stimulation emission depletion nanoscopy

Yongtao Liu[1,2*], Shihui Wen[2], Fan Wang[3], Chao Zuo[1], Chaohao Chen[4], Jiajia Zhou[2*], Dayong Jin[2,5,*]

[1]Smart Computational Imaging Laboratory(SCILab), School of Electronic and Optical Engineering, Nanjing University of Science and Technology, Nanjing, Jiangsu Province 210094, China

[2]Institute for Biomedical Materials and Devices (IBMD), Faculty of Science, University of Technology Sydney, Sydney, NSW 2007, Australia

[3]School of Physics, Beihang University, Beijing, 102206, China

[4]School of Electrical and Data Engineering, Faculty of Engineering and Information Technology, University of Technology Sydney, Sydney, NSW 2007, Australia

[5]UTS-SUStech Joint Research Centre for Biomedical Materials & Devices, Southern University of Science and Technology, Shenzhen, Guangdong, 518055, PR China

*Email：Yongtao.Liu@njust.edu.cn; Jiajia.Zhou@uts.edu.au; Dayong.Jin@uts.edu.cn;

Supplement information note1: Numerical simulation of the Yb–Tm upconversion system

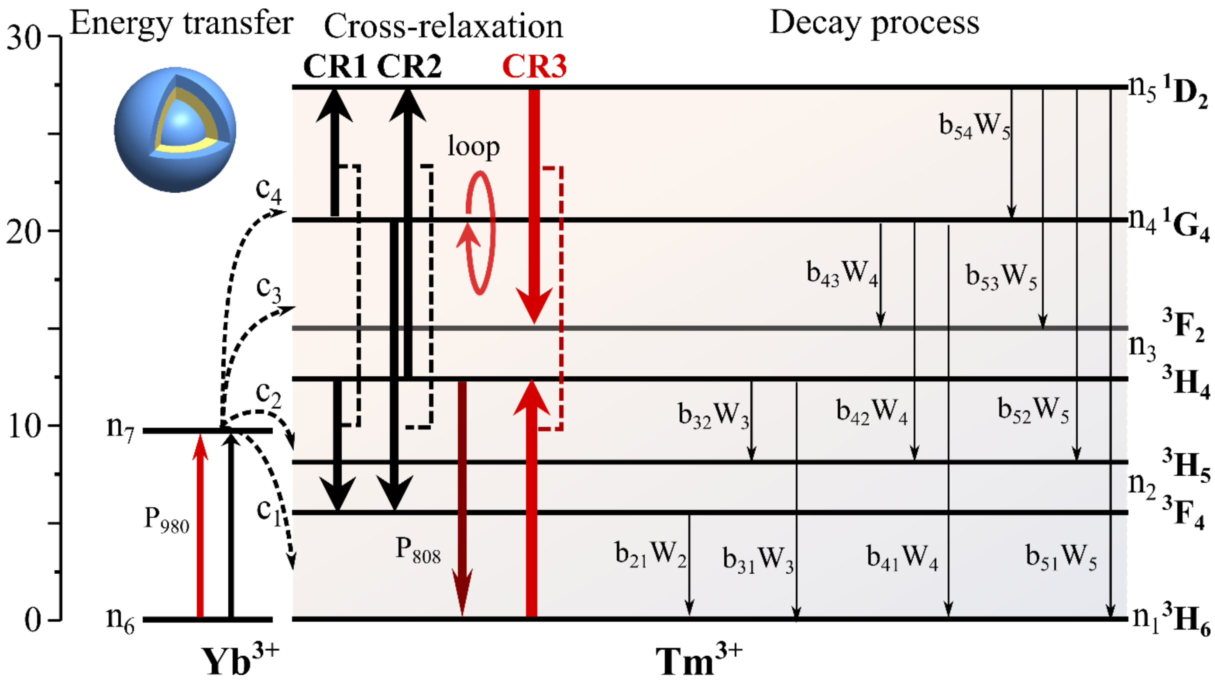

Figure S1. Energy level diagram used for rate equation modelling.

Table S1. Parameters used in rate equation simulation for the 8% Tm-doped UCNPs.

Depletion rate(s^{-1})				Decay rate(s^{-1})				
P_{980}		P_{808}	Ws	W_2	W_3		W_4	W_5
2.7×10^4		7×10^5	7.6×10^3	6.4×10^3	1.8×10^3		1.4×10^3	3×10^3
Upconversion coefficients				Cross-relaxation cofficients				
C1	C2	C3	C4	CR1		CR2		CR3
6.3×10^4	5.5×10^4	7×10^4	5×10^3	1.8×10^5		2×10^5		$4\times(10^4\text{-}10^5)$
Branching ratios								
b_{31}	b_{32}	b_{41}	b_{42}	b_{43}	b_{51}	b_{52}	b_{53}	b_{54}

0.27	0.73	0.18	0.24	0.58	0.24	0.23	0.20	0.33

The rate equations of each energy state are derived as follows:

$$\frac{dn_2}{dt} = c_1 n_1 n_{S2} - c_2 n_2 n_{S2} - w_2 n_2 + b_{32} w_3 n_3 + b_{42} w_4 n_4 + b_{52} w_5 n_5 + k_{43} n_4 n_3 + k_{34} n_3 n_4 \quad (1)$$

$$\frac{dn_3}{dt} = P_{808}(n_1 - n_3) + c_2 n_2 n_{S2} - c_3 n_3 n_{S2} - w_3 n_3 + b_{43} w_4 n_4 + b_{53} w_5 n_5 - k_{43} n_4 n_3 - k_{34} n_3 n_4 + 2k_{51} n_1 n_5 \quad (2)$$

$$\frac{dn_4}{dt} = c_3 n_3 n_{S2} - c_4 n_4 n_{S2} - w_4 n_4 + b_{54} w_5 n_5 - k_{41} n_4 n_3 \quad (3)$$

$$\frac{dn_5}{dt} = c_4 n_4 n_{s2} - w_5 n_5 - k_{51} n_1 n_5 \quad (4)$$

$$\frac{dn_{s2}}{dt} = P_{980} n_{S1} - W_S n_{S2} - (c_1 n_1 + c_2 n_2 + c_3 n_3 + c_4 n_4) n_{S2} \quad (5)$$

$$n_{S1} + n_{S2} = 1 \quad (6)$$

$$n_1 + n_2 + n_3 + n_4 + n_5 = 1 \quad (7)$$

Here, the energy of $^2F_{7/2}$ and $^2F_{5/2}$ in Yb^{3+} represented as S_1 and S_2, respectively, the energy level of $^3H_6$, $^3H_5$/$^3F_4$ and $^3F_{2,3}$/$^3H_4$, $^1G_4$, $^1D_4$ in Tm^{3+} represented as 1, 2, 3, 4, and 5, respectively. n_i (i=1-5) represent the population densities, c_i (i=1,2,3,4) is the upconversion coefficient between excited Yb^{3+} and Tm^{3+} on level i; b_{ij} is the branch ratio for the radiative transition from the initial state i to the terminal state j of Tm^{3+}, k_{ij} is the cross-relaxation coefficient. W_i is the intrinsic decay rate of Tm^{3+} on level i; where P_{980} is the absorption rate of Yb^{3+}, P_{808} is depletion ratio of Tm^{3+} ($P = \sigma\lambda I/hc$, in which λ is the excitation wavelength, I is the excitation intensity at 976/808nm, σ is the absorption cross-section of Yb^{3+} at 976/808nm, h is Planck's constant, and c is the speed of light); CR1, CR2, and CR3 refer to cross-relaxation processes that occur between ($^1G_4$, $^3H_4$), ($^1G_4$, $^3H_4$), and ($^1D_2$, $^3H_6$) states at both the ground and upper excited levels, leading to population distribution of ($^1D_2$,$^3F_4$), ($^3F_4$,$^1D_2$), and ($^3F_2$,$^3H_4$), respectively. Cross relaxation in upconversion nanoparticles is a process by which the energy from one excited ion is transferred to another nearby ion, resulting in the generation of multiple photons at a lower energy than the initial excitation. This process can occur between different energy levels of the same ion or between different ions in the nanoparticle. Cross relaxation is an important mechanism for achieving efficient upconversion luminescence in nanoparticles and plays a critical role in many applications such as bioimaging, sensing, and photodynamic therapy.

Supplement information note2: Optical light path of STED

We achieved the stimulated emission depletion(STED) microscopy based on a conventional scanning confocal system. Figure S2 provides a schematic illustration of an UCNPs STED nanoscopy system. In the present implementation of UCNPs STED nanoscopy, a single doughnut beam of 808nm laser that generated by a Vector phase plane in the focal region is co-aligned to the Gaussian focal excitation spot of 980nm. The optical system was built on a confocal scanning facility that employed a 3-axis closed-loop piezo nanopositioner(Mad city Lab). A single-mode fibre-coupled 980nm diode

laser (BL976-PAGn900, controller CLD1015, Thorlabs, maximum output power 900 mW) was used as the excitation source. One objective lens was used as the collimation lens(Thorlabs RMS10X), after collimation, the excitation beam was transmitted through the first half-wave plate(HWP, WPH05M-980, Thorlabs). And then the 4f optical system is applied with two 300mm lenses. The excitation beam was transmitted through a long-pass dichroic mirror(937 long pass filter), then reflected by a second short-pass dichroic mirror (T750spxrxt, Chroma), and the excitation beam was transmitted through the first quarter-wave plate (QWP, WPH05M-980, Thorlabs). and focused through an oil-immersion objective(Olympus; 100×, NA = 1.4)onto the sample slide. A similar setup for 808nm depletion laser light path. RMS10X lens was used as the collimation lens, and then half-wave plate and PBS were used two maintain the polarization purity. A vortex phase plate(VPP-1a, RPC Photonics) was inserted in the 808nm beam path before the 4f optical system. Then, the long-pass filter is allowed 808nm laser to go through and merge with a 980nm beam. The luminescence signal from the sample was collected by the oil-immersion objective lens. The excitation and emission were split by the 750nm short-pass filter. Before coupling the emission into a multi-mode fibre, there has a flip mirror that can transfer the emission into the camera. The end of the multi-mode fibre was connected to a single-photon avalanche diode(APD, SPCM-AQRH-14-FC, Excelitas). To select the emission from upconversion nanoparticles, the bandpass filter was inserted into the emission light path. By using the vortex phase plate, a doughnut-shaped PSF was generated at the focal plane. In the dual-laser STED system, two beams of 980nm and 808nm were carefully aligned to ensure the precise overlapping of their PSFs in both x-y and z directions. The inset figure shows us the laser pattern at different conditions, a and b show the scanning results of upconversion in XY and YZ plane by 980nm laser excitation. After the fine adjustment by the pattern of emission intensity. The precise overlapped dual laser was achieved. And then VPP was inserted into the depletion light pass to generate a doughnut shape with the fine adjustment of the quarter-wave plane as shown in the inset c and d; it shows the scanning results of upconversion in XY and YZ plane by 808nm laser excitation.

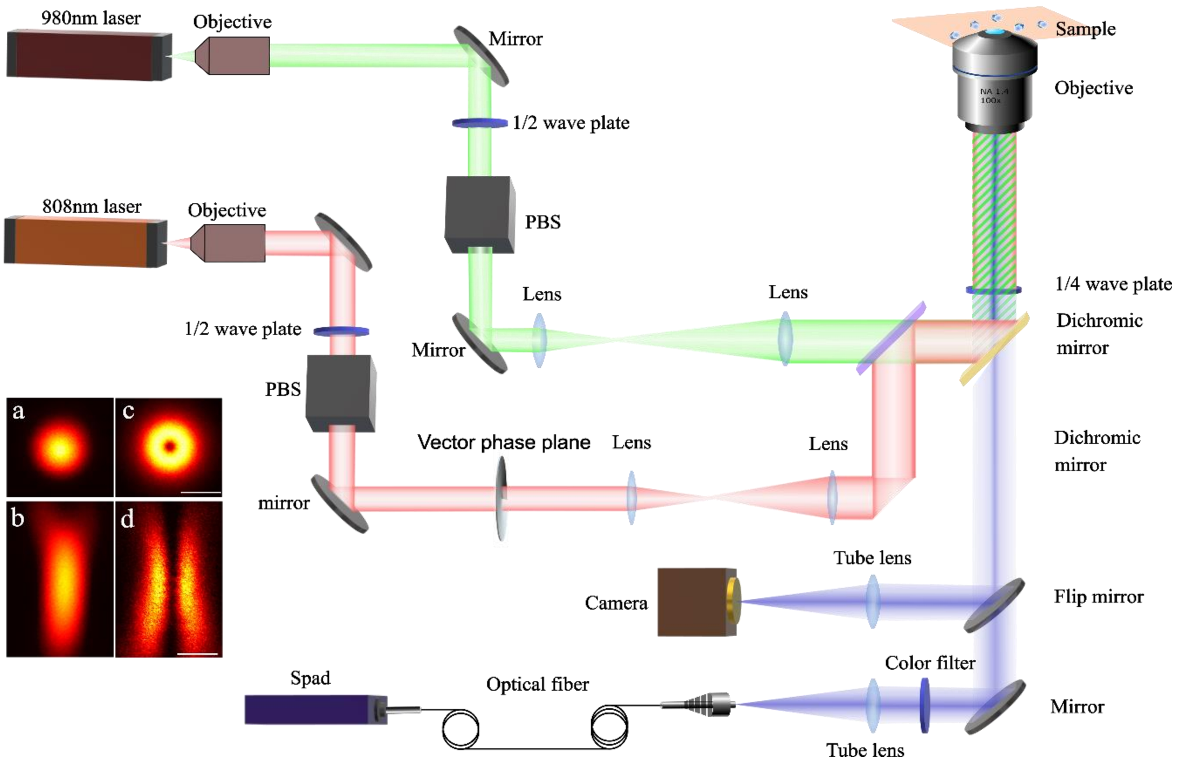

Figure S2. Optical setup of the dual-laser super-resolution microscope for probing $NaYF_4$: Yb, Tm nanocrystals. The inset (a,b,c,d) shows the cross-section scanning results of upconversion in the XY and YZ plane. a and b got from the 980nm laser excitation, c and d got from the 808nm laser excitation. Scale bar is 500nm.

Supplement information note3: PSF under different excitation conditions

To investigate the relationship between excitation power and resolution of STED, a series of confocal and STED imaging was carried out using different levels of excitation power. As shown in Figure S2, we keep depletion power at 3.4 $MW/cm^{2,}$ and then excitation power in STED scanning gradually falls from 5 mW to 1.1 mW. Prior to STED scanning, one confocal image was recorded as depicted in Figure S3, and then the depletion beam was supplied to deplete the emission from emitters. As can be seen, the full-width half-maximum (FWHM) of STED PSF is limited shrunk by the depletion beam when the excitation power is 5 mW. It confirms that at the excitation power closed to the saturation power of excitation, and the depletion efficiency is not high enough to deplete the emission pattern to achieve the resolution under the diffractive limit. When the lower excitation power was applied to the confocal and STED, it is obvious that the FWHM of the confocal and STED had a significant decrease. The non-saturation excitation of the confocal will induce higher resolution imaging. When the smaller PSF of the confocal occurs, a higher resolution of STED will be achieved. With 1.1mW excitation power, the FWHM of STED can reach 40.6 nm.

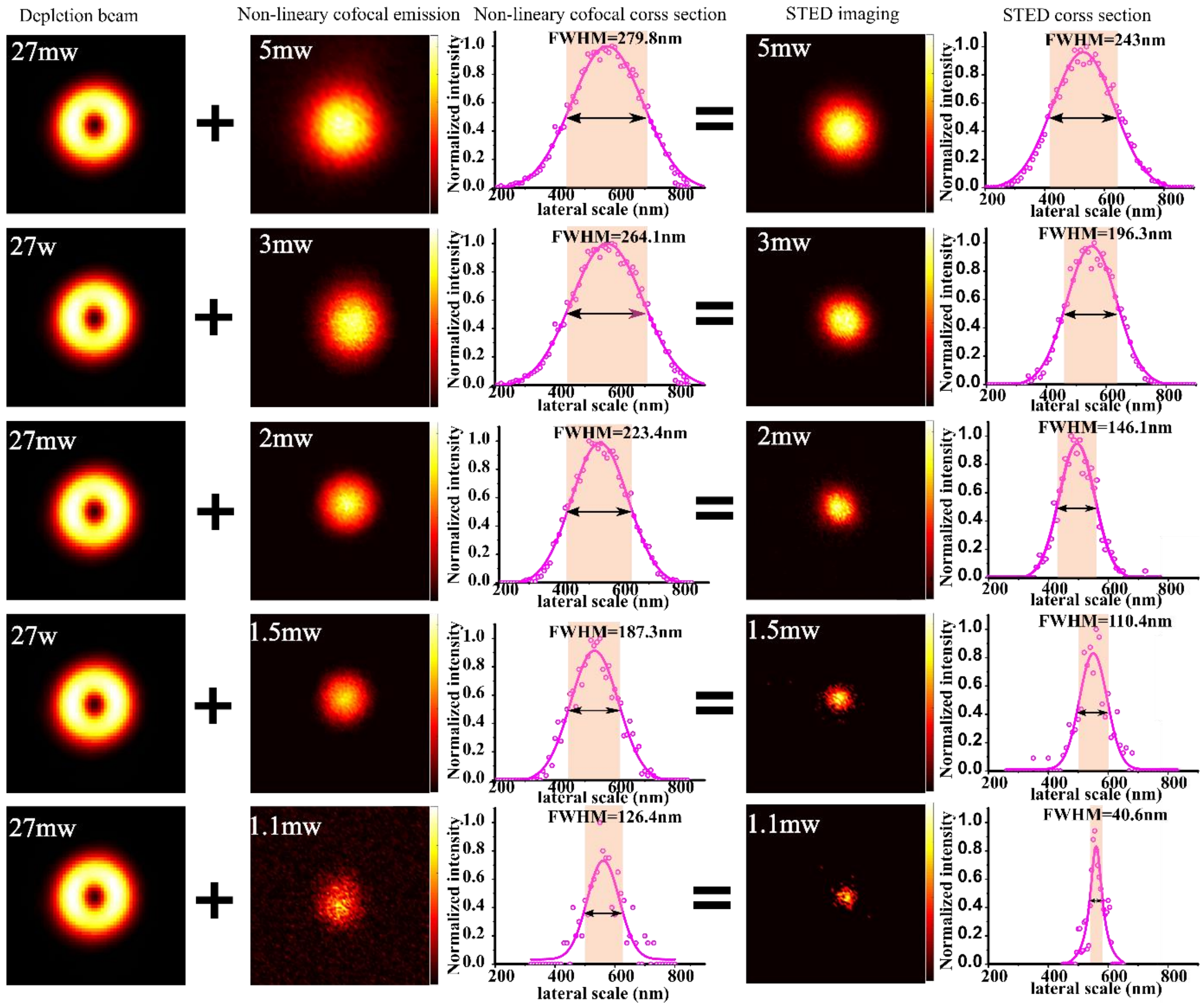

Figure S3. The confocal imaging of UCNP and its cross-section shrinking into the diffractive limit processing under the ultralow non-saturate excitation power. Based on this non-saturated excitation, the upconversion nanoparticles can achieve the highest resolution with the assistance of fixed depletion power. The cross-section of UCNPs got from confocal and STED image.

Supplement information note4: Emitter core/core-shell structure modulation at the depletion process

To discuss the core-shell structure for the enhancement of depletion efficiency, core and core-shell structure UCNPs were synthesized with the same Tm^{3+} doping concentration of 12 mol%. Under dual-laser illumination, the optical depletion ratio of 455 nm emission was measured as a function of the 808nm intensity (the results were normalized by the emission intensity). As shown in Figure S4, with the same excitation power of 980 nm laser, the core structure of UCNPs has a lower depletion power and higher depletion efficiency, which demonstrates our assumption. We further investigate the different excitation power effects with core and core-shell structures. 1 mW excitation power has a much lower depletion threshold and higher depletion efficiency than 4 mW and 8 mW, which further

exhibits in our previous experiment. The lower excitation power, the lower depletion threshold, and the higher depletion efficiency from the STED system.

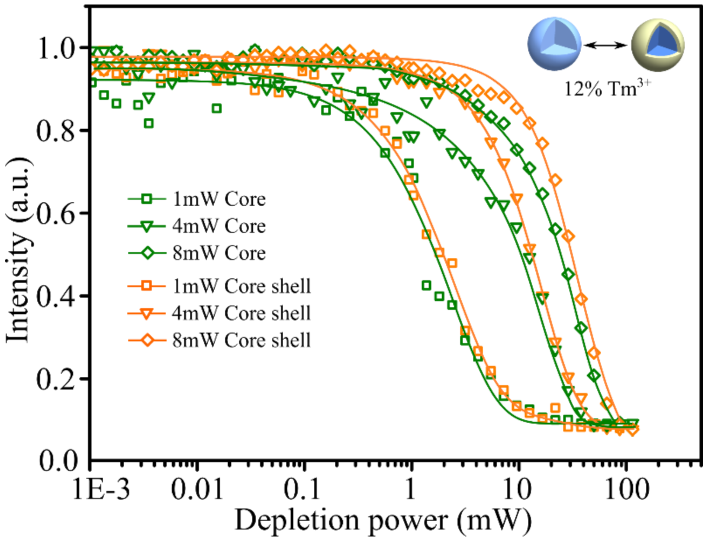

Figure S4. The depletion efficiency of core and core-shell structure nanoparticles. The results got from 12% Tm^{3+} doped core and core-shell nanoparticles.

Supplement information note5: Synthesis of upconversion nanoparticles (UCNPs)

$NaYF_4$:20%Yb,8%Tm nanocrystals were synthesized according to our previously reported method[1,2]. In a typical experiment, 1 mmol $LnCl_3·6H_2O$ (Ln=Y, Yb, Tm) with the molar ratio of 72:20:8 were added to a flask containing 6 mL OA and 15 mL ODE. The mixture was heated to 160 °C under argon flow for 30 min to obtain a clear solution and then cooled down to about 50 °C, followed by the addition of 5 mL methanol solution of NH_4F (4 mmol) and NaOH (2.5 mmol). After stirring for 30 min, the solution was heated to 80 °C under argon flow for 20 min to expel methanol, and then the solution was further heated to 300 °C for another 90 min. Finally, the reaction solution was cooled down to room temperature. The products were precipitated by ethanol and centrifuged (9000 rpm for 5 min), then washed 3 times with cyclohexane, ethanol and methanol to get the nanoparticles.

To get the nanoparticles with bigger size, layer-by-layer epitaxial growth has been employed. The shell precursors preparation is similar to that for the core nanoparticles synthesis until the step where the reaction solution was slowly heated to 150°C and kept for 20 min. Instead of further heating to 300 °C to trigger nanocrystal growth, the solution was cooled down to room temperature to yield the shell precursors. For epitaxial growth, 20 mg as-prepared core nanocrystals were added to a containing 6 ml OA and 6 ml ODE. The mixture was heated to 170 °C under argon for 30 min, and then further heated to 300 °C. Next, 0.15 ml as prepared shell precursors were injected into the reaction mixture and ripened at 300 °C for 2.5 min, followed by the same injection and ripening cycles for several times to get the nanocrystals with the desired size. Finally, the slurry was cooled

down to room temperature and the formed nanocrystals were purified according to the same procedure used for the core nanocrystals.

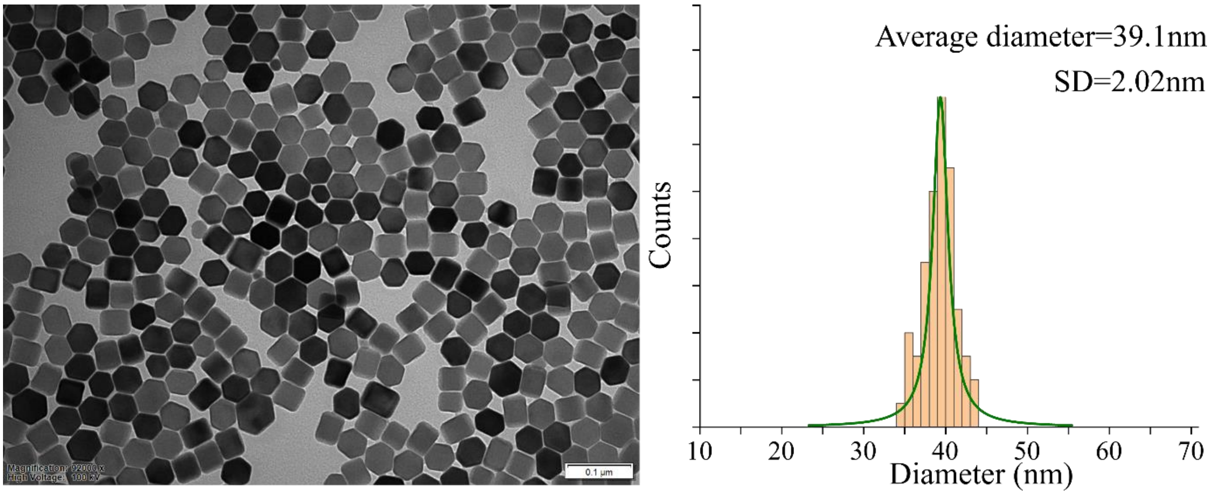

Figure S5. TEM images (left) and size distribution histograms (right) of the nanoparticles. $NaYF_4$:20%Yb,8%Tm.

Characterization Techniques. The morphology of the formed materials was characterized viatransmission electron microscopy (TEM) imaging(Philips CM10 TEM with Olympus Sis Megaview G2 Digital Camera) with an operating voltage of 100 kV. The samples were prepared by placing a drop of a dilute suspension of nanocrystals onto copper grids.

References

(1) Liu, D.; Xu, X.; Du, Y.; Qin, X.; Zhang, Y.; Ma, C.; Wen, S.; Ren, W.; Goldys, E. M.; Piper, J. A.; Dou, S.; Liu, X.; Jin, D. Three-Dimensional Controlled Growth of Monodisperse Sub-50 Nm Heterogeneous Nanocrystals. *Nat. Commun.* **2016**, 1–8. https://doi.org/10.1038/ncomms10254.

(2) Liu, Y.; Lu, Y.; Yang, X.; Zheng, X.; Wen, S.; Wang, F.; Vidal, X.; Zhao, J.; Liu, D.; Zhou, Z.; Ma, C.; Zhou, J.; Piper, J. A.; Xi, P.; Jin, D. Amplified Stimulated Emission in Upconversion Nanoparticles for Super-Resolution Nanoscopy. *Nature* **2017**, *543* (7644), 229–233. https://doi.org/10.1038/nature21366.
